# Huang Lian Jie Du decoction attenuated colitis via suppressing the macrophage Csf1r/Src pathway and modulating gut microbiota

**DOI:** 10.3389/fimmu.2024.1375781

**Published:** 2024-09-26

**Authors:** Shan Su, Ting Liu, Jia-Yi Zheng, Hai-Cui Wu, Vincent W. Keng, Shi-Jie Zhang, Xiao-Xiao Li

**Affiliations:** ^1^ State Key Laboratory of Traditional Chinese Medicine Syndrome, The Second Affiliated Hospital of Guangzhou University of Chinese Medicine, Guangzhou, China; ^2^ Department of Neurology, The Second Affiliated Hospital of Guangzhou University of Chinese Medicine, Guangzhou, China; ^3^ Department of Neurology, Guangdong Provincial Hospital of Chinese Medicine, Guangzhou, China; ^4^ Department of Pharmacy, Shenzhen Children’s Hospital, Shenzhen, China; ^5^ Department of Food Science and Nutrition, The Hong Kong Polytechnic University, Hong Kong, Hong Kong SAR, China; ^6^ Department of Applied Biology and Chemical Technology, The Hong Kong Polytechnic University, Hong Kong, Hong Kong SAR, China; ^7^ State Key Laboratory of Chemical Biology and Drug Discovery, The Hong Kong Polytechnic University, Hong Kong, Hong Kong SAR, China; ^8^ Research Center for Chinese Medicine Innovation, The Hong Kong Polytechnic University, Hong Kong, Hong Kong SAR, China; ^9^ State Key Laboratory of Chinese Medicine and Molecular Pharmacology (Incubation), The Hong Kong Polytechnic University Shenzhen Research Institute, Shenzhen, China

**Keywords:** colitis, macrophage, Csf1r, Src, gut microbiota

## Abstract

**Introduction:**

Ulcerative colitis, a subtype of chronic inflammatory bowel disease (IBD), is characterized by relapsing colonic inflammation and ulcers. The traditional Chinese herbal formulation Huang Lian Jie Du (HLJD) decoction is used clinically to treat diarrhea and colitis. However, the mechanisms associated with the effects of treatment remain unclear. This study aims to elucidate the molecular mechanistic effects of HLJD formulation on colitis.

**Methods:**

Chronic colitis in mice was induced by adding 1% dextran sulfate sodium (DSS) to their drinking water continuously for 8 weeks, and HLJD decoction at the doses of 2 and 4 g/kg was administered orally to mice daily from the second week until experimental endpoint. Stool consistency scores, blood stool scores, and body weights were recorded weekly. Disease activity index (DAI) was determined before necropsy, where colon tissues were collected for biochemical analyses. In addition, the fecal microbiome of treated mice was characterized using 16S rRNA amplicon sequencing.

**Results:**

HLJD decoction at doses of 2 and 4 g/kg relieved DSS-induced chronic colitis in mice by suppressing inflammation through compromised macrophage activity in colonic tissues associated with the colony-stimulating factor 1 receptor (Csf1r)/Src pathway. Furthermore, the HLJD formula could modify the gut microbiota profile by decreasing the abundance of *Bacteroides*, *Odoribacter*, *Clostridium_sensu_stricto_1*, and *Parasutterella*. In addition, close correlations between DAI, colon length, spleen weight, and gut microbiota were identified.

**Discussion:**

Our findings revealed that the HLJD formula attenuated DSS-induced chronic colitis by reducing inflammation via Csf1r/Src-mediated macrophage infiltration, as well as modulating the gut microbiota profile.

## Introduction

Ulcerative colitis (UC) is a subtype of inflammatory bowel disease (IBD), characterized by mucosal inflammation and bloody stool in the rectum and colon. The incidence of UC has been rising worldwide since 1990, with Europe and North America having the highest prevalence (1). Patients with UC also have a higher risk of developing colitis-associated colorectal cancer (2). The pathogenesis of UC remains unclear, with genetic susceptibility, environmental influence, dysregulated immune activity, epithelial damage, and dysbiosis of gut microbiota known to be key factors. Sustained macrophage infiltration generates a large number of inflammatory cytokines, which is the driving factor for both acute and chronic colitis (3). Clinical treatment aims to reduce inflammation, control diarrhea, and relieve pain discomfort using glucocorticoids, azathioprine, Janus kinase (JAK) inhibitors, and calcineurin inhibitors (4). Clinically, 5-aminosalicylic acid-based drugs and anti-inflammatory steroids are given to patients with moderate to severe UC (5). In addition, immunosuppressants can also be used to limit inflammation and suppress the immune response. However, anti-inflammatory steroid drugs and immunosuppressants can cause serious side effects with long-term treatment (6, 7). Traditional Chinese medicine (TCM) is an alternative for UC treatment, displaying better clinical efficacy and less toxicity (8).

The Huang Lian Jie Du (HLJD) formulation consists of four herbs—*Coptis chinensis* Franch., *Scutellaria baicalensis* Georgi, *Phellodendron chinense* Schneid., and *Gardenia jasminoides* Ellis—at a ratio of 3:2:2:3. This formulation is known for its anti-inflammatory, anti-oxidant, and anti-pyretic effects, which has been used traditionally in China for more than a millennium for the treatment of sepsis, diabetes, Alzheimer’s disease, and gastrointestinal diseases (9–11). A previous study demonstrated that HLJD decoction could ameliorate acute UC by regulating nuclear factor of kappa light polypeptide gene enhancer in B cells 1, p105 (NFKB1), and nuclear factor (erythroid-derived 2)-like 2 (NFE2L2, also known as Nrf2) signaling pathways (12). In addition, the HLJD decoction could regulate arachidonic acid and glycerophospholipid metabolism to alleviate UC in mice (13). In the last decade, HLJD decoction has been used to treat UC in China, but the underlying genetic mechanisms remain to be elucidated.

Macrophages, a key component of the innate immune response, functions as a coordinator in regulating intestinal microenvironment homeostasis, such as intracellular infections and limiting microbiota-associated inflammatory damage (3). Macrophagic differentiation and survival are dependent on colony-stimulating factor 1 (Csf1), serving as a ligand of Csf1r (14). Csf1r-dependent macrophages are indispensable for the constitutive homeostatic maintenance of the intestinal crypt in adult mice, and macrophages are the only cells expressing Csf1r within intestinal crypts (15). Interestingly, macrophage depletion with clodronate inhibited the development of colitis (16). Blockade of Csf1 (17) or Csf1r (18) resulted in inhibition of dextran sulfate sodium (DSS)-induced colitis. Therefore, Csf1r inhibitors are currently being introduced for IBD treatment. Moreover, enhanced Src, downstream of Csf1r, may not only work as a biomarker for UC (19), but may serve as promising therapeutic targets for UC treatment (20). Csf1 stimulation resulted in activation of Src kinases, which have been demonstrated to be associated with ligand-activated Csf1r (21). These studies suggest that the Csf1r/Src pathway may provide a promising therapeutic target for the treatment of UC. A recent study demonstrated the regulation of macrophage-dependent self-renewal of intestinal stem cells by gut microbiota, revealing the crosstalk between microbiota and macrophages in UC development (22). Fecal microbiota transplantation is believed to be a therapeutic option for patients with UC (23). However, the complex interactive relationships among microbiota, macrophages, and colon damage requires further extensive investigation.

In this study, we demonstrated that HLJD decoction attenuated chronic colitis in DSS-induced mice. HLJD decoction was capable of suppressing inflammation and macrophage infiltration and modulating the microbiome by affecting the Csf1r/Src pathway.

## Materials and methods

### Reagents

DSS (36 to 50 kD) was from MP Biomedicals company (160110, CA, USA). The H&E staining kit was from Solarbio (G1120, China). The total RNA kit was from Omega Bio-Tek (GA, USA). PrimeScript RT Master Mix was from Takara (RR036A, Tokyo, Japan). Forget-Me-Not™ qPCR Master Mixes were from Biotium (CA, USA). Antibodies for Csf1r (sc-46662), Src (sc-8056), p-Src (sc-166860), GAPDH (sc-32233), and ACTB (sc-47778) were from Santa Cruz (CA, USA). Antibody for F4/80 (GTX41205) was from GeneTex (CA, USA). Goat anti-rabbit IgG (H+L)-HRP conjugate (#1706515) and goat anti-mouse IgG (H+L)-HRP conjugate (#1706516) were from Bio-Rad (Hercules, CA, USA). Alexa Fluor^®^ 647 AffiniPure Goat Anti-Rat IgG (H+L) was from Jackson ImmunoResearch (Pennsylvania, USA). Antifade Mounting Medium with DAPI (#P0131) was ordered from Beyotime Biotechnology (Shanghai, China). The Metal Enhanced DAB Substrate Kit was from Thermo Fisher Scientific (MA, USA). The Stool DNA Isolation Kit (27600) was from Norgen Biotek (Ontario, Canada). Cell Counting Kit-8 (CCK8, GK10001) was from GlpBio (CA, USA). Phorbol 12-myristate 13-acetate (PMA, HY-18739) was from MedChemExpress (NJ, USA). Fetal bovine serum (FBS, 10270106), penicillin–streptomycin (10,000 U/mL, 15140122), and 0.25%Trypsin-EDTA (25200–72) were from Gibco (NY, USA). PES filter (0.22 μm; SLGP33RB) was from Millipore (MA, USA). LPS (L6529) was ordered from Sigma (Wicklow, Ireland). All reagents and kits purchased were used according to the manufacturers’ recommendations.

### Preparation of HLJD extraction and the identification of main components

*C. chinensis* Franch., *S. baicalensis* Georgi, *P. chinense* Schneid., and *G. jasminoides* Ellis were purchased from Henan Qianfang Pharmaceutical Industry Company (China). Preparation of the HLJD extract was conducted as below. Briefly, a mixture of four herbs at a weight ratio of 3:2:2:3 was boiled using distilled water twice (1:10 w/v and 1:8 w/v). The filtrates were collected and concentrated using a rotary evaporator at 60°C, followed by freeze drying in the vacuum freeze dryer. The main components of HLJD extracts were identified using Ultra-Performance Liquid Chromatography Quadrupole-Time-of-Flight Mass Spectrometry (UPLC-Q-ToF-HRMS) as described in our accepted paper (24).

### Colitis induction and HLJD treatment

C57BL/6J mice (male, approximately 6 weeks old) were obtained from Charles River (Zhejiang, China). Mice were kept under specific pathogen-free conditions in a 12-h light–dark cycle with standard rodent chow and drinking water *ad libitum*. All animals were housed and received humane care at The Hong Kong Polytechnic University Shenzhen Research Institute Centralized Animal Facility. Mice were allowed to acclimatize to the environment for 2 weeks before being randomly grouped into five groups: Normal control group (NC), HLJD group (2 g/kg, H), DSS-induced colitis group (DSS), DSS+HLJD-low dosage (2 g/kg, DHL), DSS+HLJD-high dosage (4 g/kg, DHH). DSS-induced colitis was triggered by the following protocol: 1% (w/v) DSS in drinking water for 5 days, replaced with normal drinking water for 2 days—this cycle was repeated for 8 weeks. From the second week, HLJD (low, 2 g/kg and high, 4 g/kg) was administrated by oral gavage. HLJD dosage was selected based on published papers (25, 26). At the experimental endpoint, face samples from each mouse were collected. When mice were sacrificed, colon and spleen samples were isolated for further analyses.

### Disease activity index

Mice from each group were observed and recorded weekly for body weight changes, stool blood, and stool consistency scores. Disease activity index (DAI) were calculated as previously described (27).

### H&E staining and histological assessment

H&E staining was performed using both the distal and proximal end of colon tissues according to the manufacturer’s manual. The histological assessment was conducted according to **Table 1**.

**Table 1:** Histological assessment criteria for H&E staining images of colon tissue.

Scores	Inflammation	Area
0	No inflammation	0
1	Edema or fibrosis of submucosa or erosion	1%–25%
2	Inflammatory cell infiltration limited to submucosa without ulcer	26%–50%
3	Open ulcer not reaching the proper muscle layer	51%–75%
4	Open ulcer involving the proper muscle layer but not transmural	76%–100%
5	Transmural ulcer with mild inflammatory cell infiltration	NA
6	Transmural ulcer with severe inflammation	NA

### Immunofluorescence

Paraffin sections of colon tissue (5 μm) were de-paraffinized, rehydrated, antigen retrieval solution processed, and blocked with 5% BSA at room temperature for 1 h, followed by incubation with the primary antibody of F4/80 at a dilution of 1:500 in a humidified chamber overnight at 4°C. After washing with PBST, the sections were incubated with Alexa Fluor^®^ 647 AffiniPure Goat Anti-Rat IgG (H+L) (1:500) for 2 h at room temperature, followed by PBST washing. All sections were counterstained with Antifade Mounting Medium with DAPI. Images were obtained using a Nikon confocal microscope (#FV3000, Olympus, Tokyo, Japan). Immunostaining quantification was completed using ImageJ software.

### Quantitative real-time polymerase chain reaction

Total RNA was isolated and cDNA was synthesized using the Takara RT Master Mix, followed by quantitative real-time polymerase chain reaction (qPCR) using Forget-Me-Not™ qPCR Master Mixes in a CFX96 Touch™ Real-Time PCR (Bio-Rad, CA, USA). Expression of tumor necrosis factor alpha (*Tnfa*), interleukin-6 (*Il6*), transforming growth factor beta 1 (*Tgfb*), chemokine (C-X-C motif) ligand 1 (*Cxcl1*), chemokine (C-X-C motif) ligand 10 (*Cxcl10*), chemokine (C-C motif) ligand 2 (*Ccl2*), chemokine (C-C motif) ligand 3 (*Ccl3*), *Csf1r*, *Csf1*, and interleukin 34 (*Il34*) was quantified, and the relative expression of these genes was normalized to *Actb*. Cellular expression of *CD11b*, *CD14*, *Csf1*, *Il34*, *Csf1r*, *Tnfa*, *Il6*, *iNOS*, *Ccl2*, *Tgfb*, *Arg-1*, *Vegf*, *CD206*, and *Cxcl10* was quantified and normalized to *Gapdh*. The primer sequences for mouse genes are shown in **Table 2** while the primer sequences for human genes are shown in **Table 3**.

**Table 2:** PCR primer sequences—mouse.

Name	Forward primer (5′–3′)	Reverse primer (5′–3′)
*M-Actb* (28)	AGAGCTACGAGCTGCCTGAC	AGCACTGTGTTGGCGTACAG
*M-Tnfa* (24)	CATCTTCTCAAAATTCGAGTGACAA	TGGGAGTAGACAAGGTACAACCC
*M-Il6* (24)	CCGGAGAGGAGACTTCAC	TCCACGATTTCCCAGAGA
*M-Il1b* (24)	TCCAGGATGAGGACATGAGCAC	GAACGTCACACACCAGCAGGTTA
*M-Tgfb* (29)	ATTTGGAGCCTGGACACACA	GAGCGCACAATCATGTTGGA
*M-Cxcl1* (30)	ACTGCACCCAAACCGAAGTC	TGGGGACACCTTTTAGCATCTT
*M-Cxcl10* (31)	ATGACGGGCCAGTGAGAATG	ATGATCTCAACACGTGGGCA
*M-Ccl2* (32)	TACAAGAGGATCACCAGCAGC	ACCTTAGGGCAGATGCAGTT
*M-Ccl3* (33)	TTCTCTGTACCATGACACTCTGC	CGTGGAATCTTCCGGCTGTAG
*M-Csf1r* (24)	GGACCTACCGTTGTACCGAG	CAAGAGTGGGCCGGATCTTT
*M-Csf1* (34)	GTGTCAGAACACTGTAGCCAC	TCAAAGGCAATCTGGCATGAAG
*M-Il34*	GGACTCGCCTGGCTATACTG	CTGAAGCCGGTTCTTGTACTG
*M-iNOS* (35)	GTTCTCAGCCCAACAATACAAGA	GTGGACGGGTCGATGTCAC
*M-Arg-1* (36)	TGTCCCTAATGACAGCTCCTT	GCATCCACCCAAATGACACAT
*M-CD206* (37)	CTCTGTTCAGCTATTGGACGC	TGGCACTCCCAAACATAATTTGA
*M-Vegf* (38)	GCACATAGAGAGAATGAGCTTCC	CTCCGCTCTGAACAAGGCT
*V3–V4* (39)	ACTCCTACGGGAGGCAGCAG	GGACTACHVGGGTWTCTAAT

**Table 3:** PCR primer sequences—human.

Name	Forward primer (5′–3′)	Reverse primer (5′–3′)
*H-Gapdh* (40)	GGAGCGAGATCCCTCCAAAAT	GGCTGTTGTCATACTTCTCATGG
*H-CD11b* (41)	GCCTTGACCTTATGTCATGGG	CCTGTGCTGTAGTCGCACT
*H-CD14* (42)	ACGCCAGAACCTTGTGAGC	GCATGGATCTCCACCTCTACTG
*H-Csf1* (43)	TGGCGAGCAGGAGTATCAC	AGGTCTCCATCTGACTGTCAAT
*H-Il34* (44)	CCTGGCTGCGCTATCTTGG	AGTGTTTCATGTACTGAAGTCGG
*H-Csf1r* (45)	TCCAAAACACGGGGACCTATC	CGGGCAGGGTCTTTGACATA

### Western blotting

Expression of Csf1r (1:1,000), p-Src (1:1,000), Src (1:2,000), Gapdh (1:3,000), or ACTB (1:3,000) was detected using a previously described Western blotting protocol (46). ImageJ was used to quantify the intensity of resulting bands.

### Immunohistochemical staining

Immunohistochemical (IHC) staining was performed as described previously (47). The primary Csf1r antibody and F4/80 antibody were used at a dilution of 1:200 and 1:500, respectively.

### 16S rRNA amplicon sequencing

Stool samples were collected from each mouse at the experimental endpoint. Stool DNA was isolated and quantified as previously described (27). Hypervariable regions V3–V4 were amplified using primers 338F and 806R (**Table 2**). Amplicon was sequenced using the Illumina MiSeq PE300 platform (Majorbio, China). The raw data are available on NCBI Sequence Read Archive database (SRA: PRJNA955002), with the link https://dataview.ncbi.nlm.nih.gov/object/PRJNA955002.

### Cell culture

The human leukemic cell strain THP-1 and mouse macrophage cell line RAW264.7 were obtained from Prof. Yanxiang Zhao from The Hong Kong Polytechnic University. THP-1 cells were grown in RPMI-1640 medium supplemented with 10% FBS and 1% penicillin–streptomycin. RAW264.7 cells were cultured in high-glucose DMEM medium supplemented with 10% FBS and 1% penicillin–streptomycin. Cells were kept at 37°C with 5% CO_2_.

### Cell viability assay

HLJD freeze-dried powder was dissolved in DMSO and filtered with a 0.22-μm PES filter. For the cell viability assay, THP-1 or RAW264.7 cells were seeded in a 96-well plate at a density of 3 × 10^5^ and 1 × 10^5^ cells/mL, respectively. Different doses of HLJD (3.125/6.25/12.5/25/50/100/200 µg/mL) were exposed to cells for 24 h, followed by CCK8 incubation for 1 h at 37°C with 5% CO_2_. Plates were read at 450 nm.

### THP-1 differentiation assay

To induce differentiation of monocytes THP-1 into macrophages, PMA at the concentration of 80 ng/mL were administrated to THP-1 cells (48) with or without HLJD intervention. After 24-h PMA stimulation, cells were imaged using a phase contrast microscope (ECLIPSE Ts2-FL NIKON).

### Macrophage functional assay

LPS (10 ng/mL) were exposed to RAW264.7 cells for 4 h to elicit M1 phenotype macrophage. M1 and M2 markers, as well as CSF1R-related molecules, were detected in HLJD (25 µg/mL) alone or in combination with LPS-treated cells.

### Statistical analysis

Statistical analysis was performed with GraphPad Prism v 8.4.2. Data are presented as means ± SEM. Brown–Forsythe and Welch analysis of variance (ANOVA) followed by Dunnett’s multiple comparisons test or two-way ANOVA followed by Tukey’s multiple comparisons test were used to compare the mean values as well as significant difference among groups. Certain tests were also mentioned in the figure legends. *p* < 0.05 was considered statistically significant.

## Results

### HLJD decoction ameliorated DSS-induced colitis in mice

**Figure 1:**
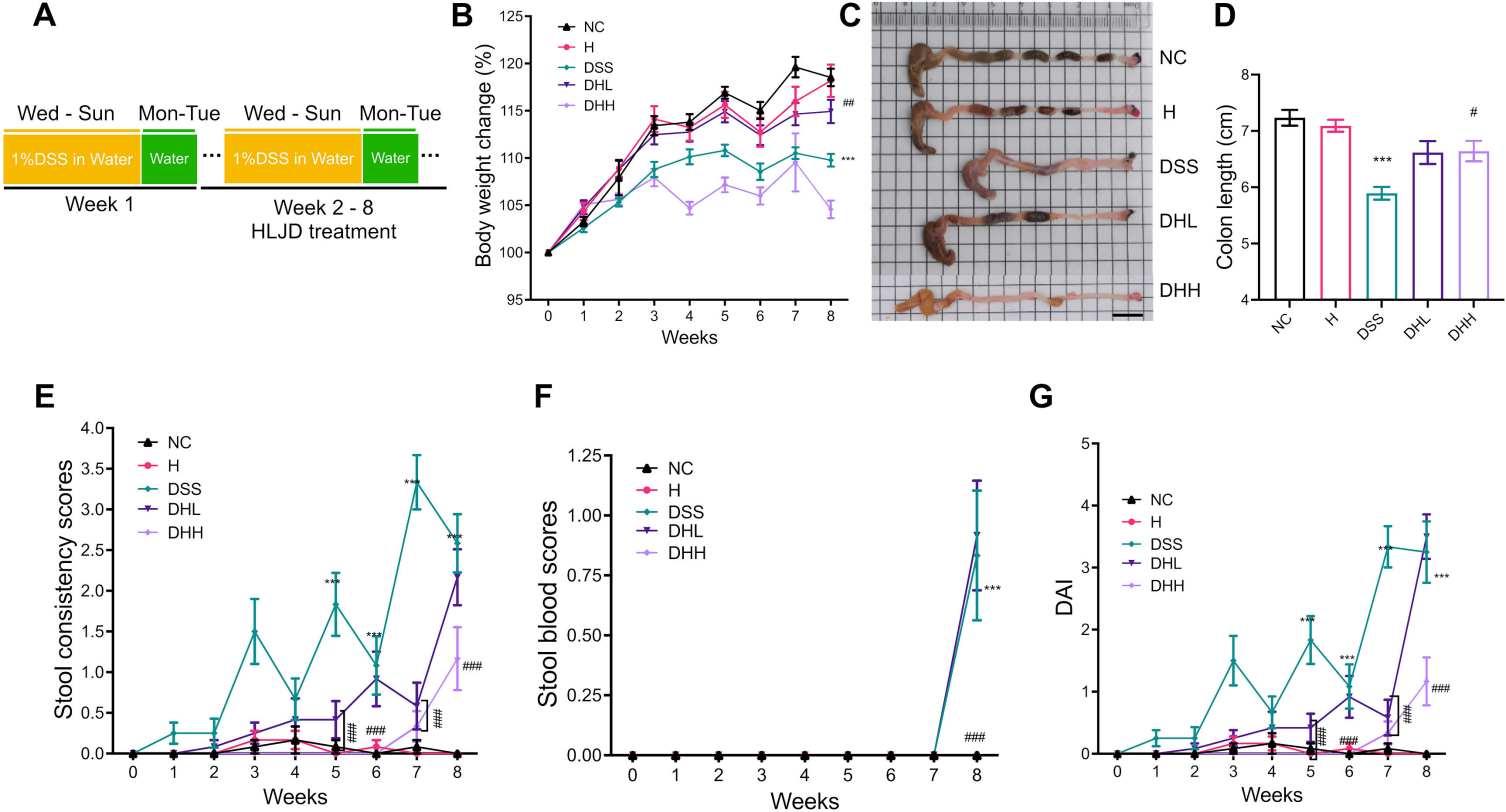
HLJD decoction improved chronic colitis conditions in the DSS-induced mouse model. **(A)** Timeline of the animal experiment. **(B)** Body weight changes among different groups (*n* = 12). (**C, D**) Representative colon samples and colon lengths measured from each group (*n* = 12). Scale bar, 1 cm. (**E, F**) Stool consistency (*n* = 12) and blood scores (*n* = 12) recorded during treatment measured from each group, respectively. **(G)** DAI scores (*n* = 12). NC, normal control group; H, HLJD at the dose of 2 g/kg daily by oral gavage; DSS, mice given 1% DSS 5 days a week for 8 weeks; DHL and DHH, mice given 1% DSS were treated with HLJD at 2 and 4 g/kg daily by oral gavage, respectively. Values were presented as means ± SEM. ***p < 0.001 *vs.* NC; ##*p* < 0.01, ###*p* < 0.001 *vs.* DSS.

To evaluate the efficacy of HLJD formulation on colitis, a chronic colitis mouse model was established using 1% DSS in drinking water continuously for 8 weeks. HLJD decoction at both low and high doses (2and 4 g/kg, respectively) were administrated from the second week (**Figure 1A**). Mice treated with HLJD alone were included to evaluate any side effects of this drug (group H). In comparison with the NC group, there was significantly less body weight gain in the DSS group, while HLJD at a low dose (DHL) had a trend to rescue the phenotype (**Figure 1B**) (*p* < 0.0001 for DSS *vs.* NC, *p* = 0.0053 for DHL compared with DSS). Consistent with our ongoing anti-obesity study, HLJD at a high dose (DHH) could decrease the body weight gain. In addition, mice have significantly shorter colons in the DSS group compared with the NC group, while this phenotype was improved in DHL and DHH groups (**Figures 1C, D**) (*p* < 0.0001 for DSS *vs.* NC, *p* = 0.0547 and 0.0232 for DHL and DHH compared with DSS). DSS-induced mice have significantly higher stool consistency scores from the fifth week compared with the NC, DHL, and DHH group mice (*p* < 0.0001 for DSS *vs.* NC from week 5 to week 8, *p* < 0.001 for DHL compared with DSS on week 5 and week 7, *p* < 0.001 for DHH compared with DSS from week 5 to week 8) (**Figure 1E**). For the stool blood scores, the DSS group demonstrated significantly higher scores at week 8 (*p* < 0.001), and only DHH mice rescued this phenotype (*p* < 0.001 for DSS *vs.* NC on week 8, *p* < 0.001 for DHH compared with DSS on week 8) (**Figure 1F**). DSS-induced mice also have significantly higher DAI scores from the fifth week compared with the NC, DHL, and DHH group mice (*p* < 0.001 for DSS *vs.* NC from week 5 to week 8, *p* < 0.001 for DHL compared with DSS on week 5 and week 7, *p* < 0.001 for DHH compared with DSS from week 5 to week 8) (**Figure 1G**). Mice from the H group showed no difference with that in the NC group in body weight changes, colon length, and stool scores. Furthermore, HLJD treatment at a dose of 4 g/kg also showed no toxic effects in mice, which are consistent with published papers (25, 26). These results demonstrated that HLJD at both dosages show the trends to ameliorate DSS-induced symptoms, and a high dose of HLJD resulted in better outcome in DSS-stimulated colitis mice compared with a low dose of HLJD.

**Figure 2:**
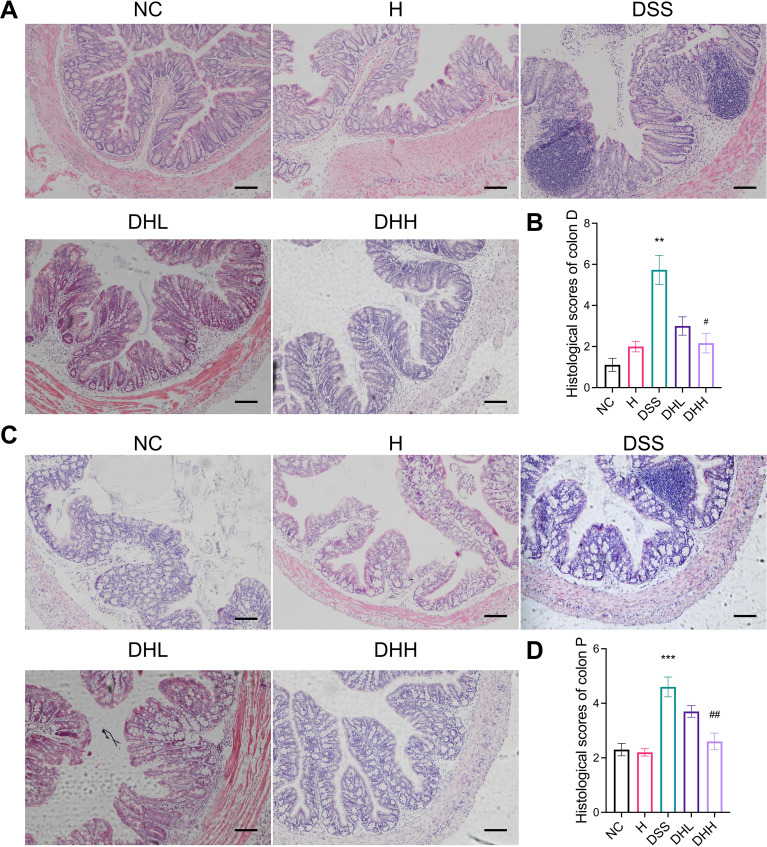
HLJD decoction attenuated colonic damage in the DSS-induced mouse model. Representative H&E staining of both distal end **(A)** and proximal end **(C)** of colon sections from different treatment groups (scale bar, 100 µm). (**B, D**) Calculated histological scores of distal ends (*n* = 6) and proximal ends (*n* = 10) of colon sections based on collected H&E images. Colon D, distal end of colon tissue; Colon P, proximal end of colon tissue. NC, normal control group; H, HLJD at the dose of 2 g/kg daily by oral gavage; DSS, mice given 1% DSS 5 days a week for 8 weeks; DHL and DHH, mice given 1% DSS were treated with HLJD at 2 and 4 g/kg daily by oral gavage, respectively. Data were expressed as mean ± SEM. **p < 0.01, ***p < 0.001 *vs.* NC; #*p* < 0.05, ##*p* < 0.01 *vs.* DSS.

As shown in **Figure 2A**, distal ends of colonic tissue in NC and H group mice have intact mucosa, submucosa, and muscle layer. In contrast, DSS group mice were characterized by inflammatory cell infiltration, thickened mucosal layers, and crypt loss, resulting in significantly increased histological scores (*p* = 0.0046 for DSS *vs.* NC, *p* = 0.0868 and 0.0202 for DHL and DHH compared with DSS, respectively) (**Figure 2B**). Both doses of HLJD were able to partly restore crypt structures and inhibited inflammation (**Figures 2A, B**). Similarly, proximal ends of colonic tissue in DSS mice demonstrated severe inflammation with larger inflammatory area compared with that in NC mice (**Figure 2C**). DHH attenuated this phenotype and significantly decreased the histological scores but not in DHL mice (*p* = 0.0008 for DSS *vs.* NC, *p* = 0.3512 and 0.0056 for DHL and DHH compared with DSS, respectively) (**Figures 2C, D**), indicating that a high dose of HLJD could compromise the damage induced by DSS in both distal and proximal ends of the colon. The criteria for the histological assessment are shown in **Table 1**.

### HLJD decoction suppressed macrophage infiltration and inflammation in mice

**Figure 3:**
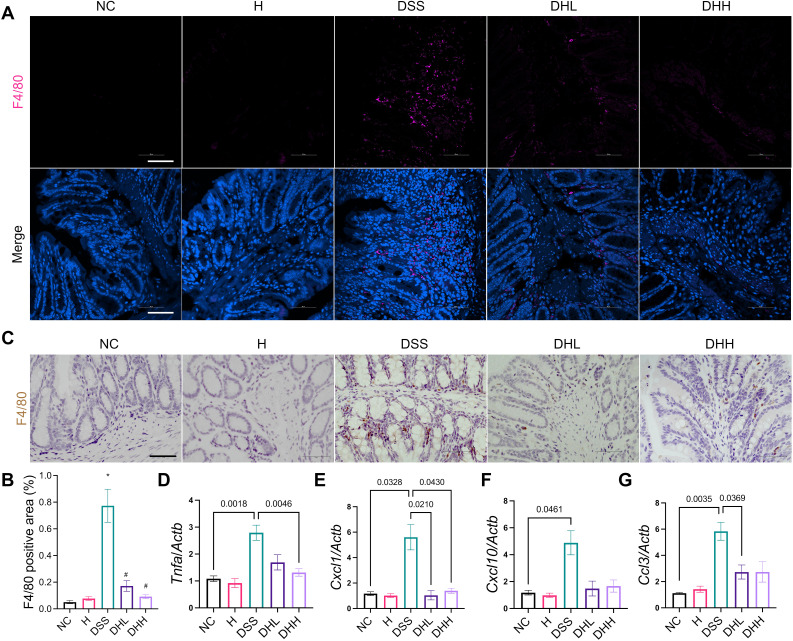
HLJD decoction ameliorated colonic inflammation in the DSS-induced chronic colitis mouse model. **(A)** Representative immunofluorescent staining images of F4/80 in the distal end of colon tissues from each group. Scale bar, 50 µm. **(B)** Quantification of F4/80-positive macrophages in the colon (*n* = 6). **(C)** Representative IHC staining images of F4/80 in the distal end of colon tissues from each group. Scale bar, 50 µm. (**D–G**) Relative mRNA expression of *Tnfa*, *Cxcl1*, *Cxcl10*, and *Ccl3* normalized to *Actb* (*n* = 7). NC, normal control group; H, HLJD at the dose of 2 g/kg daily by oral gavage; DSS, mice given 1% DSS 5 days a week for 8 weeks; DHL and DHH, mice given 1% DSS were treated with HLJD at 2 and 4 g/kg daily by oral gavage, respectively. Data were expressed as mean ± SEM. *p < 0.05 *vs.* NC; #*p* < 0.05 *vs.* DSS.

During chronic colitis, homeostatic macrophages are massively replenished by classic monocytes and their macrophage progeny, which will migrate to inflamed mucosa in response to chemokines including CCL2, CCL7, and CCL8 (49). These monocyte-derived macrophages express inducible nitric oxide synthase (iNOS) and generate large amounts of IL-1β, TNF, IL-6, and IL-12, working as a rich source of cytokines and chemokines in the inflamed tissue (50, 51). Our data demonstrated that DSS-induced mice have significantly increased macrophage infiltration in the distal end of colon tissue, shown as increased F4/80-positive cells (**Figure 3A**). HLJD significantly reverted this phenotype (*p* = 0.0145 for DSS *vs.* NC, *p* = 0.0261 and 0.0188 for DHL and DHH compared with DSS, respectively) (**Figures 3A, B**). A similar result was obtained in IHC staining of F4/80-labeled macrophages in the colonic tissues of mice from each group (**Figure 3C**). Furthermore, HLJD (50 µg/mL) reduced PMA-induced cells attaching without affecting cell viability (**Supplementary Figures S1A, B**), indicating that HLJD may inhibit differentiation of monocytes to macrophages. This was supported by the decreased expression of the macrophage marker CD11b in PMA-HLJD50-treated cells compared with that in PMA-treated cells (*p* = 0.0134 for PMA vs. CON, *p* = 0.0425 for PMA-HLJD50 compared with PMA) (**Supplementary Figure S1C**). However, the monocyte marker CD14 was not significantly changed between PMA-HLJD50- and PMA-treated cells (**Supplementary Figure S1D**).

Expression of cytokines and chemokines in colon tissues was examined by qPCR. *Tnfa* was significantly increased in DSS-treated mice compared with NC, and DHH significantly reverted this trend (**Figure 3D**). There was a trend of higher expression levels of *Il6*, *Tgfb*, and *Ccl2* in DSS mice compared with NC, while HLJD treatment tend to reduce their expression without statistical significance (**Supplementary Figures S2A–C**). The activated macrophages could secrete C-X-C motif ligand 1 (CXCL1) (52) and CXCL10 (53), which are chemoattractant chemokines that recruit neutrophils in response to inflammation. *Cxcl1* was higher in the DSS group than in the NC group (*p* = 0.0328), which was significantly decreased in HLJD-treated mice at both doses (*p* = 0.0210 and *p* = 0.0430 for DHL and DHH compared with DSS, respectively) (**Figure 3E**). Similarly, for *Cxcl10*, expression levels in DSS-treated mice were significantly higher than in the NC group (*p* = 0.0461), but DHL and DHH showed trends of reduction (**Figure 3F**). In addition, expression of neutrophil recruitment genes *Ccl3* was significantly increased in DSS-treated mice compared with NC (*p* = 0.0035), and DHL, but not DHH, could significantly reduce *Ccl3* expression level when compared with DSS mice (*p* = 0.0369) (**Figure 3G**). DSS-induced mice had remarkably increased spleen weight percentages, while HLJD significantly reverted this phenotype (*p* = 0.0003 for DSS vs. NC, *p* = 0.0001 and 0.0010 for DHL and DHH compared with DSS, respectively) (**Supplementary Figure S2D**), further highlighting the anti-inflammatory role of HLJD in DSS-induced colitis. Consistent result was obtained using 10 ng/mL of the LPS-stimulated RAW264.7 cellular model. HLJD at the concentration of 100 µg/mL exerted no inhibitory effects on RAW264.7 cells (**Supplementary Figure S3A**). Expression of M1 macrophage markers, including *Tnfa*, *Il6*, and *iNOS*, was reduced at the mRNA level in cells treated with LPS and HLJD (25 µg/mL) compared with that in LPS-triggered cells (*p* = 0.0437, *p* = 0.0549, and *p* = 0.0491, respectively, for LPS-HLJD25 vs. LPS) (**Supplementary Figure S3B**). However, HLJD treatment could not affect M2 phenotype markers (**Supplementary Figure S3C**). Level of chemokines *Cxcl10* and *Ccl2* showed a trend to increase after LPS induction, but there was no significant difference (**Supplementary Figures S3D, E**).

### HLJD decoction inhibited the Csf1r/Src pathway

**Figure 4:**
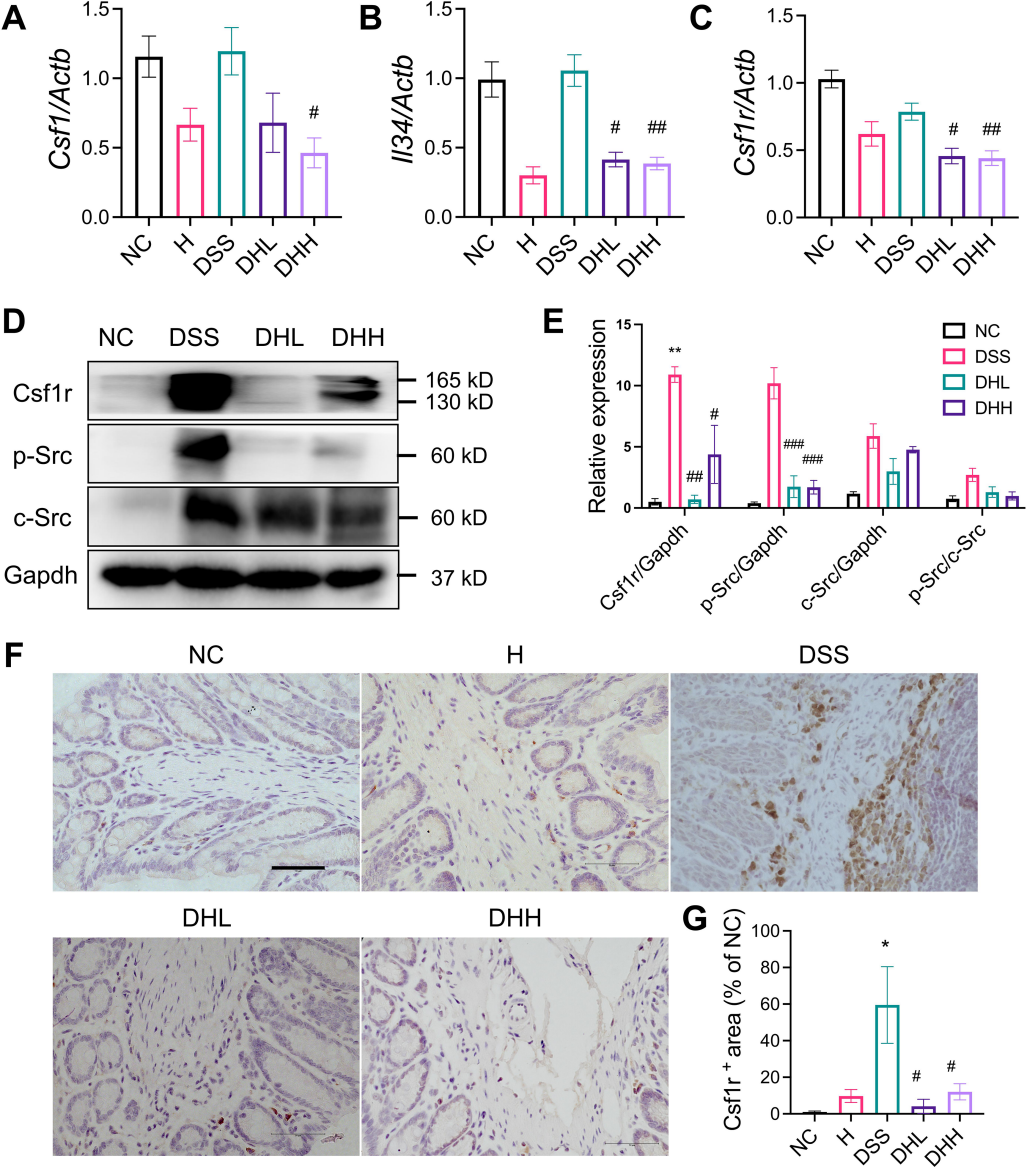
HLJD decoction inhibited Csf1r/Src levels in the DSS-induced chronic colitis mouse model. (**A** to **C**) Relative mRNA expression of *Csf1* (*n* = 6), *Il34* (*n* = 6), and *Csf1r* (*n* = 9) normalized to *Actb*. **(D)** The representative Western blot image of Csf1R, p-Src, Src, and Gapdh. **(E)** Quantitative analyses of blots (*n* = 3). **(F)** Representative IHC staining image of Csf1r in colon tissues taken from each group. Scale bar, 50 µm. (**G**) Quantification of Csf1r expression in the colon (*n* = 3). NC, normal control group; H, HLJD at the dose of 2 g/kg daily by oral gavage; DSS, mice given 1% DSS 5 days a week for 8 weeks; DHL and DHH, mice given 1% DSS were treated with HLJD at 2 and 4 g/kg daily by oral gavage, respectively. Data were expressed as mean ± SEM. *p < 0.05, **p < 0.01 *vs.* NC; #*p* < 0.05, ##*p* < 0.01, ###*p* < 0.001 *vs.* DSS.

In order to further clarify the molecular mechanism(s) underlying the effects of HLJD decoction on macrophage-regulated inflammation conditions in DSS-induced chronic colitis, we evaluated expression of the Csf1r/Src pathway, which has been reported to be a key player in contributing to mucosal inflammation during colitis development (18). The mRNA expression levels of Csf1r ligands *Csf1* and *Il34* (54) remained unchanged in the colon of DSS-treated mice (**Figure 4A, B**). Although there was a trend for reduced *Csf1* level in the DHL-treated group, *Il34* expression was significantly reduced. Both *Csf1* and *Il34* were significantly decreased in DHH-treated mice (*p* = 0.0388 and *p* = 0.0074, respectively) (**Figures 4A, B**). *Csf1r* expression was significantly lower in DHL- and DHH-treated mice (*p* = 0.0108 and *p* = 0.0068, respectively) (**Figures 4C**). Protein expression of Csf1r was enhanced in DSS-induced mice but significantly reduced after HLJD administration. Src level was induced in mice treated with DSS and no significant changes in expression were observed with HLJD treatment. However, elevated phosphorylated Src (p-Src) in DSS mice was significantly reduced in DHL and DHH mice (*p < 0.05, **p < 0.01, ***p < 0.001 vs. NC; #*p* < 0.05, ##*p* < 0.01, ###*p* < 0.001 vs. DSS) (**Figures 4D, E**). The expression of Csf1r among groups was further confirmed by IHC assay (**Figure 4F**), as the expression level of Csf1r was increased in DSS-treated mice and reduced as a result of HLJD treatment (*p* = 0.0003 for DSS vs. NC, *p* = 0.0001 and 0.0010 for DHL and DHH compared with DSS, respectively) (**Figure 4G**). CSF1R regulates the differentiation of most circulating macrophages through CSF1 and IL34 (55). Intriguingly, levels of *Csf1*, *Il34*, and *Csf1r* increased in PMA-induced THP-1 cells (*p* = 0.0567, *p* = 0.0448, and *p* = 0.0474, respectively, for PMA *vs.* CON), while additional HLJD treatment tend to reverse these alterations (*p* = 0.3805, *p* = 0.1499, and *p* = 0.4583, respectively, for PMA vs. CON) (**Supplementary Figures S1E–G**), indicating that HLJD has a trend to inhibit the CSF1R signaling pathway in macrophage differentiation triggered by PMA. Consistently, *Csf1* was enhanced when RAW264.7 cells were triggered by LPS (*p* = 0.0064), but this trend was inhibited after treatment with HLJD (**Supplementary Figure S3F**). Expression of *Il34* and *Csf1r* remains unchanged among groups (**Supplementary Figures S3G, H**).

**Figure 5:**
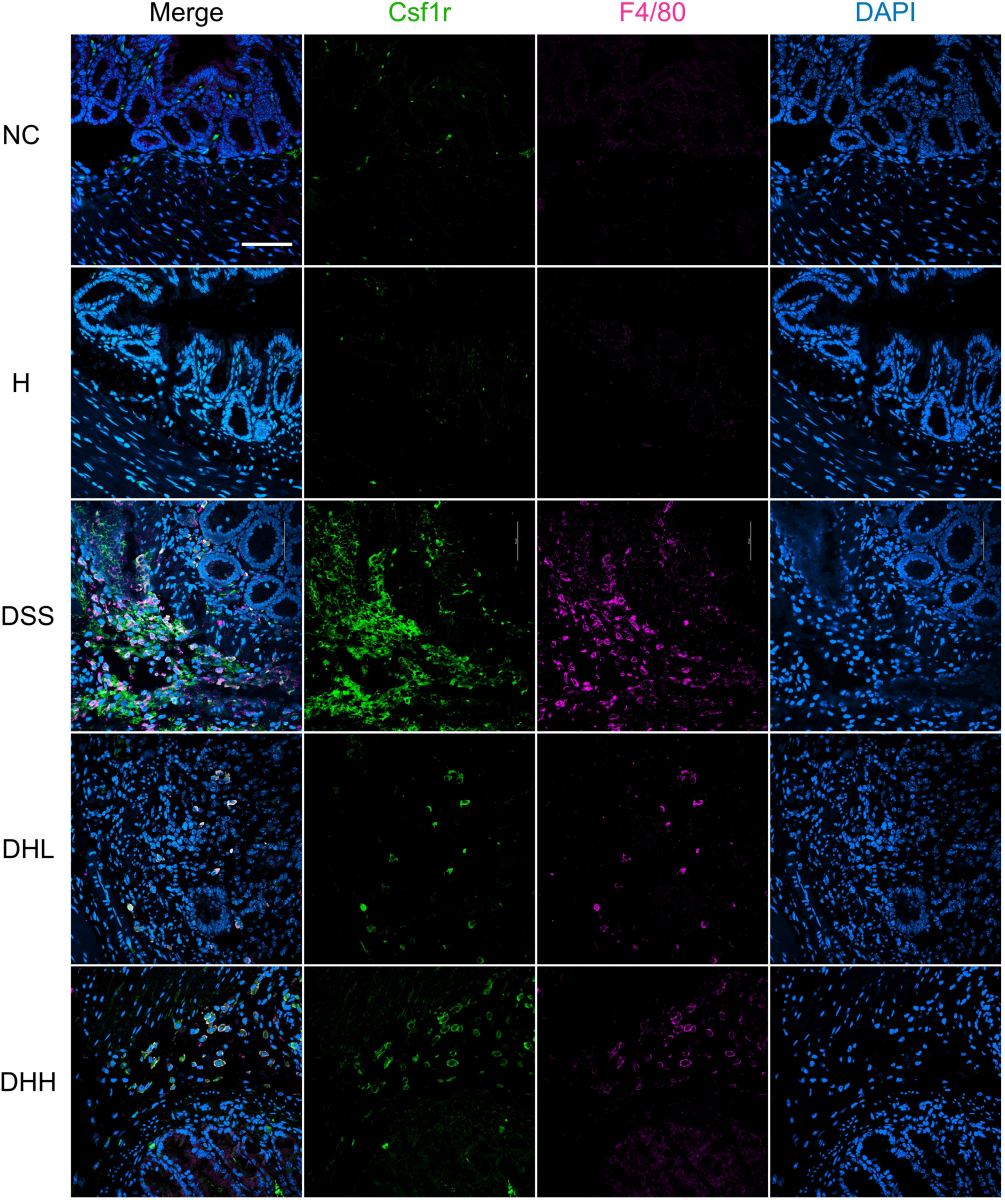
HLJD decoction inhibited Csf1r levels in F4/80-positive macrophages of the DSS-induced chronic colitis mouse model. Scale bar, 50 µm. NC, normal control group; H, HLJD at the dose of 2 g/kg daily by oral gavage; DSS, mice given 1% DSS 5 days a week for 8 weeks; DHL and DHH, mice given 1% DSS were treated with HLJD at 2 and 4 g/kg daily by oral gavage, respectively (*n* = 5).

To further confirm that Csf1r expression on macrophages was inhibited after HLJD treatment, F4/80 and Csf1r were co-labeled to show their expression and distributions using a confocal microscope imaging system. As shown in **Figure 5**, expression of F4/80 and Csf1r was obviously expressed higher in DSS-induced colon tissue, which was compromised after HLJD administration. Colocalization of F4/80 and Csf1r was observed after DSS induction.

### Effects of HLJD decoction on the gut microbiota of DSS-treated mice

**Figure 6:**
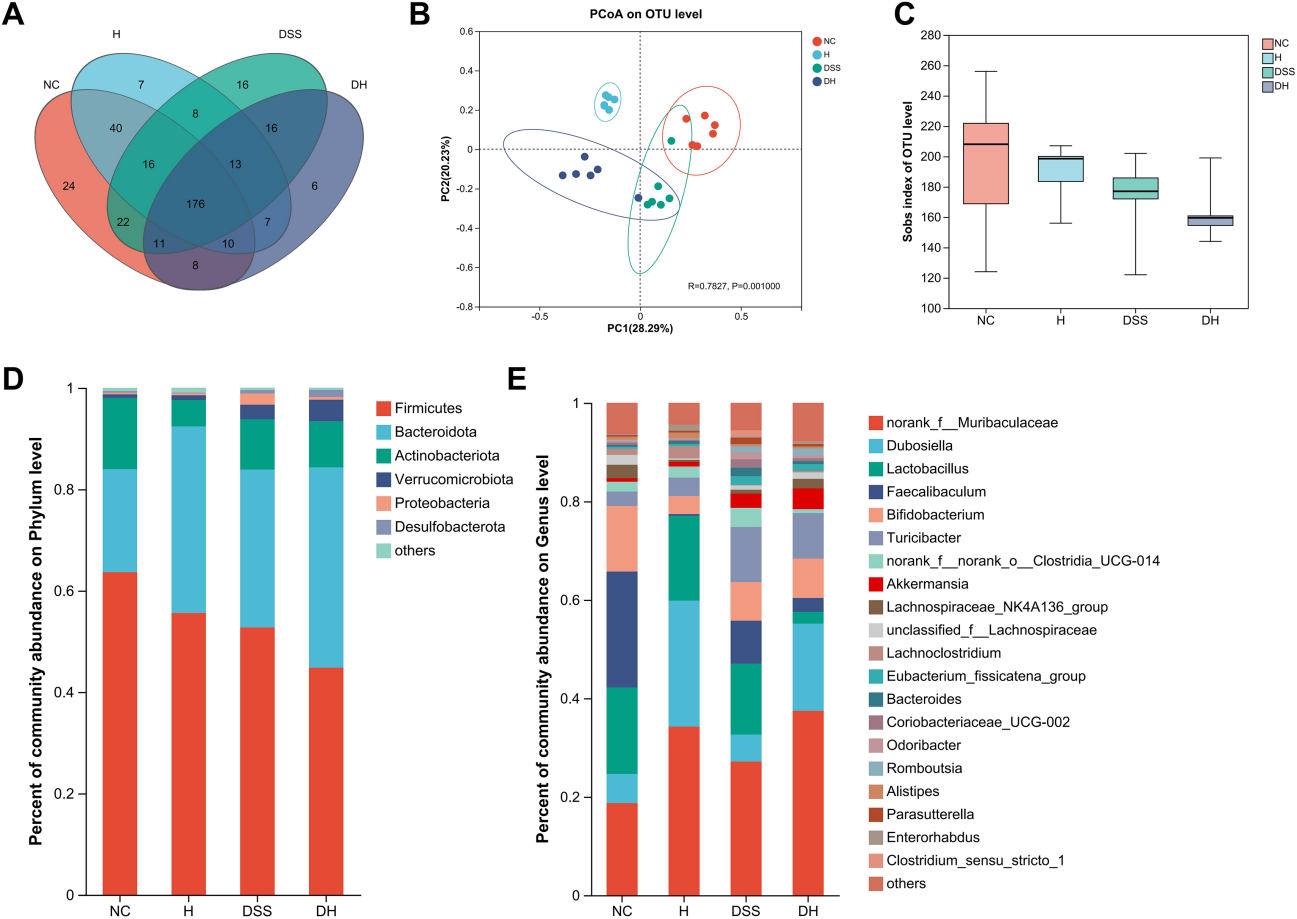
Alterations in the gut microbiota composition from different groups. **(A)** The Venn diagram representing the operational taxonomic units (OTUs) in samples obtained from different groups. **(B)** PCoA of the bacterial community composition based on Bray–Curtis distances. Dots represent individual samples. **(C)** Sobs index of the OTU level. (**D, E**) Abundant phyla and genera found among groups, respectively (*n* = 6). NC, normal control group; H, HLJD at the dose of 2 g/kg daily by oral gavage; DSS, mice given 1% DSS 5 days a week for 8 weeks; DH, mice given 1% DSS were treated with HLJD at 2 g/kg by oral gavage.

**Figure 7:**
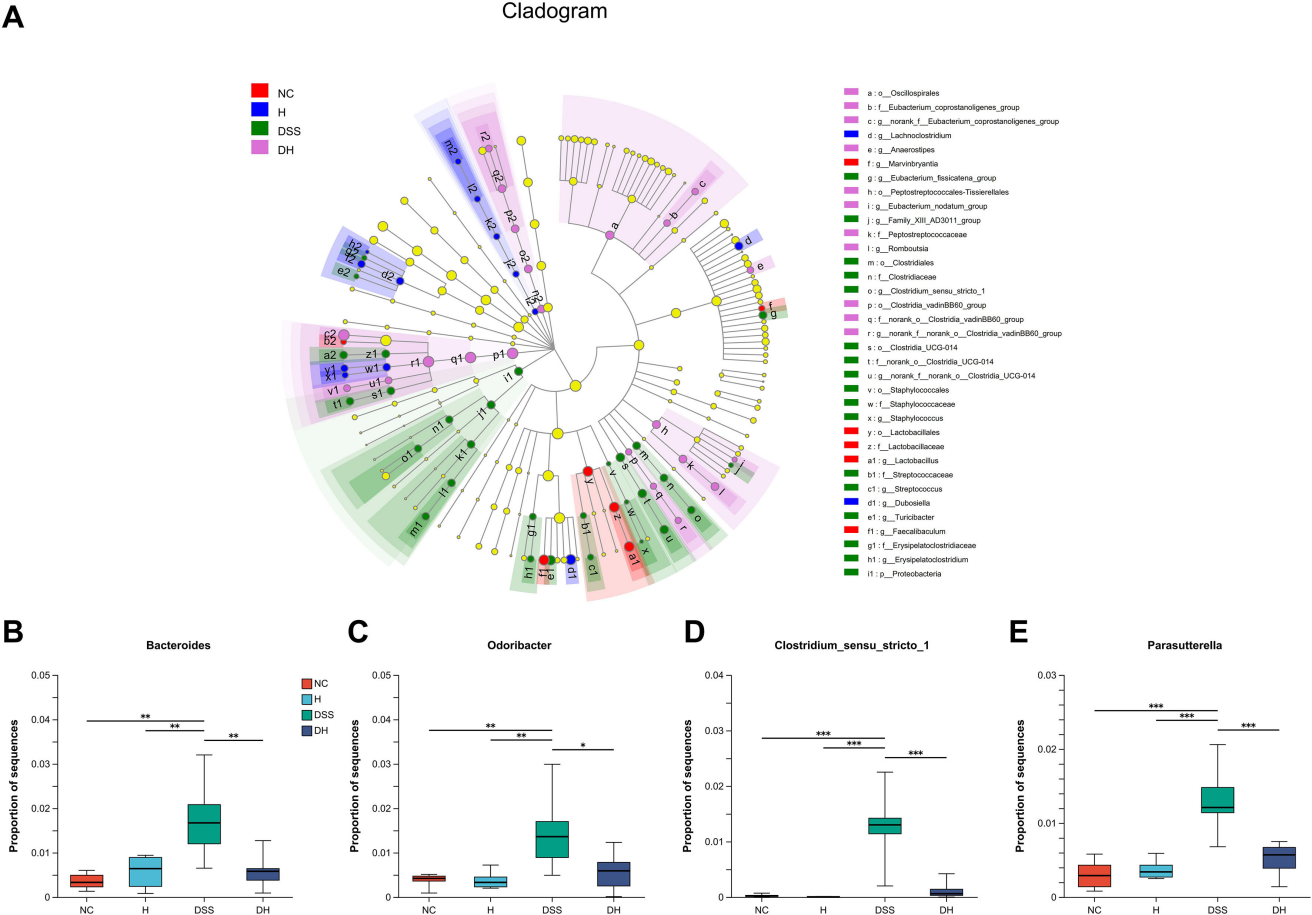
Cladogram of the LEfSe analysis of the gut microbiota composition in different groups. **(A)** The microbial compositions were compared at different evolutionary levels. (**B–E**) Comparison of relative abundant microbes at the genus level in different groups. NC, normal control group; H, HLJD at the dose of 2 g/kg daily by oral gavage; DSS, mice given 1% DSS 5 days a week for 8 weeks; DH, mice given 1% DSS were treated with HLJD at 2 g/kg by oral gavage. Data were expressed as mean ± SEM (*n* = 6). *p < 0.05, **p < 0.01, ***p < 0.001 *vs.* the indicated group, Kruskal–Wallis test.

**Figure 8:**
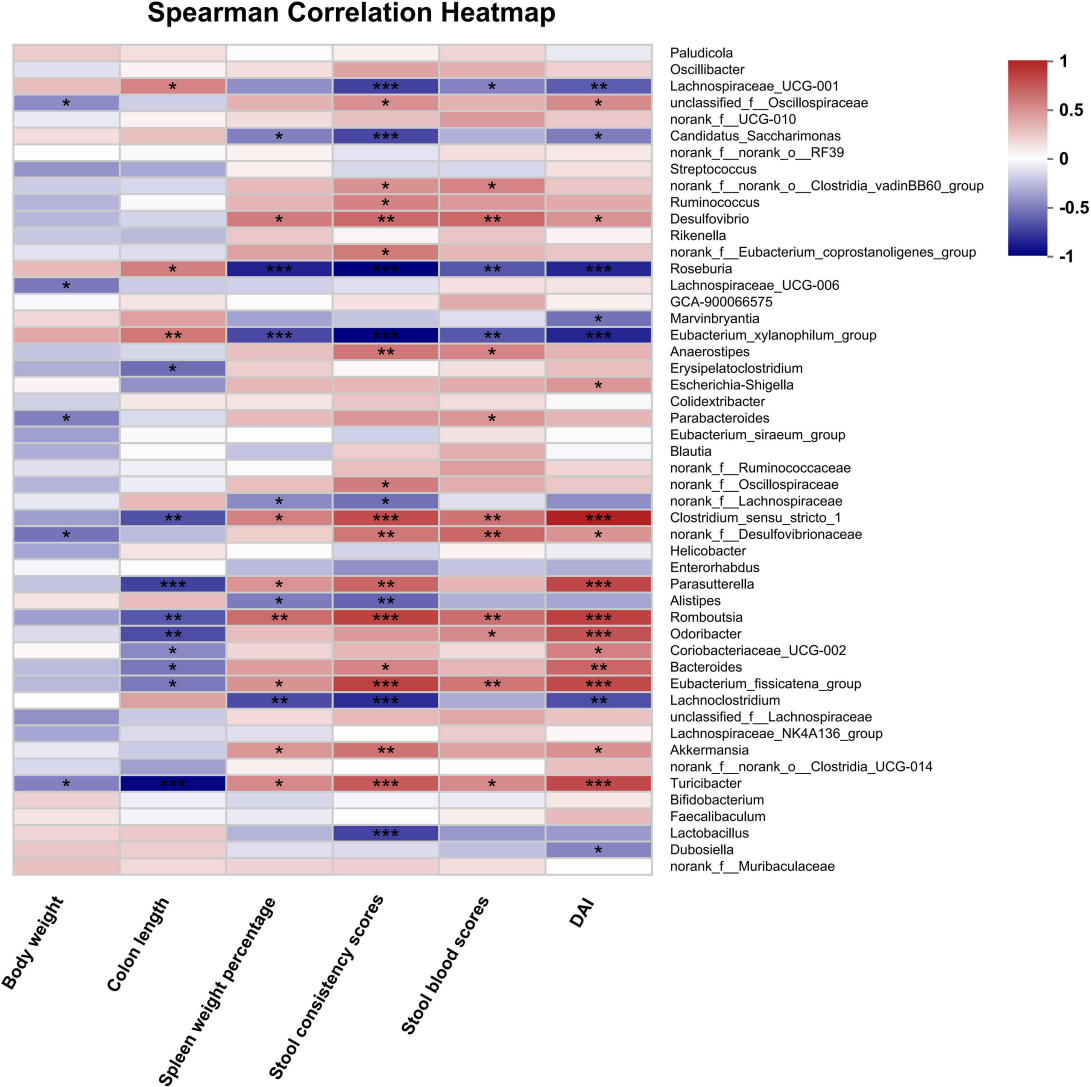
Spearman correlation analysis between disease-associated parameters and intestinal bacterial flora at the genus level after HLJD treatment. NC, normal control group; H, HLJD at the dose of 2 g/kg daily by oral gavage; DSS, mice given 1% DSS 5 days a week for 8 weeks; DH, mice given 1% DSS were treated with HLJD at 2 g/kg by oral gavage. Data were expressed as mean ± SEM (*n* = 6). *p < 0.05, **p < 0.01, ***p < 0.001 *vs.* the indicated group.

To explore the effects of HLJD intervention on the gut microbiota of DSS-induced colitis mice, the fecal samples (*n* = 6 for each group) from NC, H, DSS, and DHL (marked as DH) were analyzed by 16S rRNA sequencing. The overall differences in species numbers among each group were shown in a Venn image (**Figure 6A**). PCoA on OTU level plots indicated clustering of samples among groups (**Figure 6B**). The Sobs index of the OTU level is shown in **Figure 6C**, indicating a trend towards slightly lower community richness and evenness in DSS and DH groups. Differences in the community compositions in both phylum and genus levels were shown (**Figures 6D, E**). Moreover, LEfSe analyses were performed to identify bacterial groups that showed significant differences in different levels among groups (**Figure 7A**). As shown in **Figures 7B–E**, the abundance of *Bacteroides*, *Odoribacter*, *Clostridium*_*sensu*_*stricto*_*1*, and *Parasutterella* was dramatically enhanced in DSS mice, but was significantly suppressed after HLJD treatment (*p* = 0.009674 for *Bacteroides*; *p* = 0.01726 for *Odoribacter*; *p* = 0.0005901 for *Clostridium_sensu_stricto_1*; and *p* < 0.001for *Parasutterella*). Subsequently, the correlations between colitis symptom-related parameters such as body weight, colon length, spleen weight percentage, stool consistency scores, blood stool scores, DAI, and microbe abundance at the genus level were analyzed using Spearman correlation analyses (**Figure 8**). The abundance of *Roseburia*, *Eubacterium_xylanophilum_group*, *Clostridium_sensu_stricto_1*, *Romboutsia*, *Eubacterium_fissicatena_group*, and *Turicibacter* significantly correlated with more than five symptom-related parameters with *R-* and *p*-values shown in **Table 4**.

**Table 4:** *R* and *P* values of Spearman correlation analyses.

Species	Body weight		Colon length		Spleen weight percentage		Stool consistency scores		Stool blood scores		DAI	
	*R*-value	*p*-value	*R*-value	*p*-value	*R*-value	*p*-value	*R*-value	*p*-value	*R*-value	*p*-value	*R*-value	*p*-value
g_Roseburia	0.25793	0.22365	0.49805	0.01326	−0.76962	0.00001	−0.88521	0	−0.58738	0.00255	−0.75523	0.00002
g_Eubacterium_xylanophilum_group	0.33546	0.10904	0.52345	0.00867	−0.6319	0.00093	−0.88499	0	−0.58723	0.00255	−0.75505	0.00002
g_Clostridium_sensu_stricto_1	−0.34235	0.10152	−0.60761	0.00164	0.49455	0.01402	0.69031	0.00019	0.54107	0.00633	0.86595	0
g_Romboutsia	−0.33819	0.10601	−0.58492	0.00268	0.57323	0.00341	0.73837	0.00004	0.56375	0.00412	0.74317	0.00003
g_Eubacterium_fissicatena_group	−0.24957	0.23957	−0.46462	0.02217	0.42174	0.0401	0.73517	0.00004	0.53479	0.00709	0.70262	0.00013
g_Turicibacter	−0.43913	0.0318	−0.86306	0	0.46	0.02372	0.64123	0.00073	0.45956	0.02387	0.70704	0.00011

## Discussion

UC is a form of IBD featured by mucosal inflammation and bloody diarrhea in the colon with currently no curable options. As a pathological process of UC, the effector immune cells such as macrophages, neutrophils, and inflammatory monocytes play important regulatory roles in the development of UC (56). There are currently few studies reporting the efficacy of HLJD decoction affecting these immune cells (12, 57). In the current study, we aim to identify the effects and molecular mechanisms of HLJD decoction on DSS-induced chronic colitis in mice.

Epithelium barrier leakage due to epithelial cell damage caused by DSS is utilized to construct the chronic colitis animal models in our study. Sulfate groups of the DSS have a negative charge, which is toxic to the colonic epithelial cells and ultimately compromise the epithelium integrity to affect its permeability. Moreover, DSS has an anti-coagulant property that can further aggravate intestinal bleeding (58). Therefore, DSS is widely used to induce chronic and acute colitis for the establishment of UC animal models and assessment of UC drugs. In this study, chronic colitis was induced by administrating eight repeated cycles of 1% DSS and water; each cycle involves DSS administration for 5 days followed by water for 2 days. This DSS protocol was in line with published chronic colitis regimes (59, 60), where successful colitis animal models can be confirmed by the observation of loss of crypt architecture in the mucosa, lymphocytosis, and neutrophil infiltration (61). The body weight of DSS-treated mice increased slower in comparison with NC mice, which was different from a sharp decreased body weight in acute colitis induced by high dose of DSS as previously reported (59). There was less body weight gain in DHH mice than in DSS mice (**Figure 1B**), which is consistent with our previous observation that HLJD decoction at 4 g/kg could compromise body weight gain in high-fat diet-fed mice. However, the body weight of mice in DSS and DHH groups still increased during the experiment. Therefore, effects of DHH on body weight did not affect the calculation of DAI scores, which reflected the overall influence of body weight loss, stool consistency, and stool blood conditions. Interestingly, the stool consistency scores and DAI increased with fluctuations in DSS mice since mice were allowed access to normal water for 2 days every week, which mimics the chronic colitis with mild and sustained inflammation in the colon tissue. HLJD administration reversed the stool consistency scores and DAI scores in a dose-dependent manner (**Figures 1E, G**), indicating that HLJD treatment could improve the diarrhea condition triggered by DSS. In line with this, colon length in DHH mice was longer compared with that in DSS mice (**Figures 1C, D**). DSS-induced damage was mainly confined to the large intestines, where HLJD treatment improved the distal and proximal ends of colon tissue damage, with only a high dose of HLJD significantly ameliorating DSS-induced lesions in the colon (**Figure 2**). These observations highlight that a high dose of HLJD exerted more extensive anti-inflammatory effects along the colon tissue, which is needed for a better therapeutic outcome.

Macrophage infiltration has been demonstrated to play an important role in UC progression (62). In DSS-treated mice, the murine macrophage marker F4/80 was increased in the lamina propria of the colonic mucosa; however, less macrophage infiltration was observed in HLJD-treated mice (**Figure 3**). This improvement may result from its anti-inflammatory effects through inhibition on macrophage activity. Cytokines are key components regulating inflammatory activities during chronic UC disease progression. HLJD significantly inhibited *Tnfa* expression, demonstrating its anti-inflammatory effects upon DSS induction (**Figure 3D**). In fact, anti-TNF drugs are commonly used in patients with IBD (63), supporting the potential applications of HLJD on UC treatment. Chemokine Cxcl1 is a potent neutrophil chemoattractant (64). Blockade of Cxcl10 protects mice from acute colitis by enhancing crypt cell survival (65). CCL2 and CCL3 are chemokines promoting neutrophil accumulation in colitis, where CCL3 inhibition could compromise colitis damage (66). HLJD decoction could decrease *Tnfa* expression, as well as other chemokines involved in mediating inflammatory process. In addition, spleen weight in DSS mice was increased, as a possible result from aggregated systematic inflammation. HLJD could significantly decrease spleen weight, especially at high dose, indicating that HLJD at 2 g/kg could limit the inflammation condition, while HLJD at 4 g/kg exerted immune-suppressive effects with unknown mechanisms. A previous study has demonstrated that HLJD could ameliorate DSS-induced acute colitis by inhibiting the NF-κB pathway, activating Nrf2 signaling and enhancing intestinal barrier function (12). This study focused on the effects of HLJD on the oxidative stress provoked by DSS, and the NF-κB pathway has been demonstrated to play critical roles in the regulation of oxidative stress and associated inflammatory response. The JAK/STAT pathway mediated the downstream biological effects in response to cytokine receptor binding, a reaction occurred after aberrant cytokine release, and the JAK inhibitor has been shown to be effective in treating colitis (67). The PI3K/Akt signaling pathway may play major roles in regulating intestinal epithelial cells’ proliferation and improving the intestinal barrier injury when the intestinal barrier is disrupted by DSS in colitis (68–70), which is a different way to improve colitis. Given the fact that monocyte-derived macrophages express iNOS and generate large amounts of cytokines and chemokines in the inflamed tissue (50, 51), we wonder if HLJD could affect macrophage differentiation.

The Csf1r/Src pathway has been reported to participate in the regulation of macrophage-associated development of colitis (71). Our *in vivo* data revealed that the inflammatory condition and macrophage infiltration in the colon were suppressed after HLJD treatment. Expression of *Csf1* and *Il34*, as well as their receptor *Csf1r*, was reduced as a result of HLJD intervention (**Figures 4A, C**). The protein expression of Csf1r in the colon tissues of DSS mice was higher but was significantly reduced after HLJD treatment (**Figures 4D–G**). Discrepancies between the mRNA and protein levels of Csf1r in the DSS group may result from complicated and varied posttranscriptional mechanisms involving mRNA-to-protein translation regulation. The Src and p-Src, downstream of activated Csf1r (21), were upregulated in DSS mice, but p-Src levels were reduced after HLJD intervention (**Figure 4D**). By using a confocal microscope imaging system, Csf1r was found to be reduced after HLJD treatment in F4/80-positive macrophages in the DSS-exposed colon tissues, which further confirms that HLJD decoction could inhibit inflammatory macrophage activities in DSS-induced colitis mice (**Figure 5**). Our *in vitro* data suggested that HLJD reduced PMA-induced THP-1 cells attaching and inhibited the expression of the macrophage marker CD11b (**Supplementary Figure S1**). Csf1/Csf1r was considered to be a key regulator of macrophage proliferation and differentiation, and our result demonstrated that HLJD tends to inhibit Csf1 in PMA-induced THP-1 cells (**Supplementary Figure S1E**) and LPS-induced RAW264.7 cells (**Supplementary Figure S3F**), suggesting that HLJD may affect macrophage differentiation and polarization through the Csf1/Csf1r signaling pathway. However, our previous study demonstrated that activation of the Trem2/Dap12 signaling pathway in the lateral habenula (LHb) brain region of DSS-induced mice contributed to the colitis-associated depression, where the expression of *Csf1r* in the LHb of DSS-stimulated mice did show a trend to increase, but there was no statistical difference (24). The discrepancy in the expression of Csf1r may result from the different genetic background of the LHb brain region and colon tissue, which resulted in different genetic alterations triggered by DSS in mice. Our result was supported by a previous study reporting that blockade of Csf1 could significantly inhibit DSS-induced colitis (17), further indicating that Csf1r plays an important role in mediating intestinal mucosal inflammation, and serves as a promising target for colitis intervention.

DSS-induced symptoms were confined to the colon, specifically in the distal colon where an enormous number of microorganisms live. These microorganisms are capable of altering and producing molecules that shape the inflammatory environments and affect intestinal homeostasis (58). Our data revealed that DSS could change the microbiota profile (**Figure 6**). DSS decreased the alpha microbic diversity as reflected by the Sobs index and was further reduced by HLJD treatment (**Figure 6C**), which is consistent with a previous study demonstrating the bacteriostatic effect of HLJD treatment (72). HLJD treatment alone could change the gut microbiota profile especially for the bacteria with the highest abundance in the genus level, for example, *Muribaculaceae*, *Dubosiella*, and *Faecalibaculum*, and similar changes could be observed in the DH group (**Figure 6E**). For those most abundant bacteria, including *Dubosiella* and *Lactobacillus*, they showed similar levels between the NC and DSS group, illustrating a weak association between these bacteria and DSS-induced colitis. Interestingly, our data showed that the abundance of the genus *Bacteroides* increased in DSS mice (**Figure 7B**), which appeared to contradict a recent study demonstrating that *Bacteroides vulgatus*, one of the predominant *Bacteroides* species in the gut, attenuated experimental mice colitis (73). However, our result was consistent with a clinical study reporting that *B. vulgatus* contributes to colitis (74). Thus, the effects of *Bacteroides* remain controversial in IBD until now. The higher abundance of *Odoribacter* in DSS-induced mice was also reverted after HLJD treatment (**Figure 7C**). It has been demonstrated that the relative abundance of *Clostridium_sensu_stricto_1* (75) and *Parasutterella* (76) increased significantly in patients with IBD, while the addition of HLJD significantly reverted the gut microbiota imbalance by modifying the abundance of these specific bacteria and contributing to a prominent improvement in DSS-induced gut microbiota dysbiosis. Furthermore, significant correlations between colon length, spleen weight percentage, stool scores, DAI, and specific bacteria were observed (**Table 4**), further illustrating the potential application of HLJD intervention in the prevention of UC. These data demonstrated that HLJD decoction could modify the gut microbiota profile, which is associated with the compromised symptoms induced by DSS.

HLJD decoction, working as a classic TCM formula, has been used clinically for thousands of years, demonstrating its efficacy and safety in clinical use. The current study was the first to explore the anti-inflammatory mechanism of HLJD in terms of the macrophage activities. However, further studies are needed to explore the efficacy of candidate components in HLJD decoction as well as their underlying mechanisms in the regulation of macrophage activities and functions. The limitation of this current study lies in the lack of validation of the key roles of gut microbiota in HLJD-associated effects using germ-free mice, which is included in our future research plan.

## Conclusions

Our results demonstrated that HLJD decoction inhibited the inflammatory responses in the chronic colitis mouse model induced by DSS. This anti-inflammatory effect may result from its inhibition on the Csf1r/Src signaling pathway, as well as modifications in the gut microbiota. Our study indicated that HLJD might be a promising novel therapeutic option for the treatment of UC.

## Data Availability

The datasets presented in this study can be found in online repositories. The names of the repository/repositories and accession number(s) can be found below: https://www.ncbi.nlm.nih.gov/, SRA: PRJNA955002.
