# Supplementary material for: Huang Lian Jie Du decoction attenuated colitis via suppressing the macrophage Csf1r/Src pathway and modulating gut microbiota
Source: Front Immunol. 2024 Sep 26;15:1375781. doi: 10.3389/fimmu.2024.1375781 (PMC11464287; doi:10.3389/fimmu.2024.1375781)
Supplement: Supplementary file 1 [file DataSheet1.docx]

**Supplementary material- Figure S1-S3**


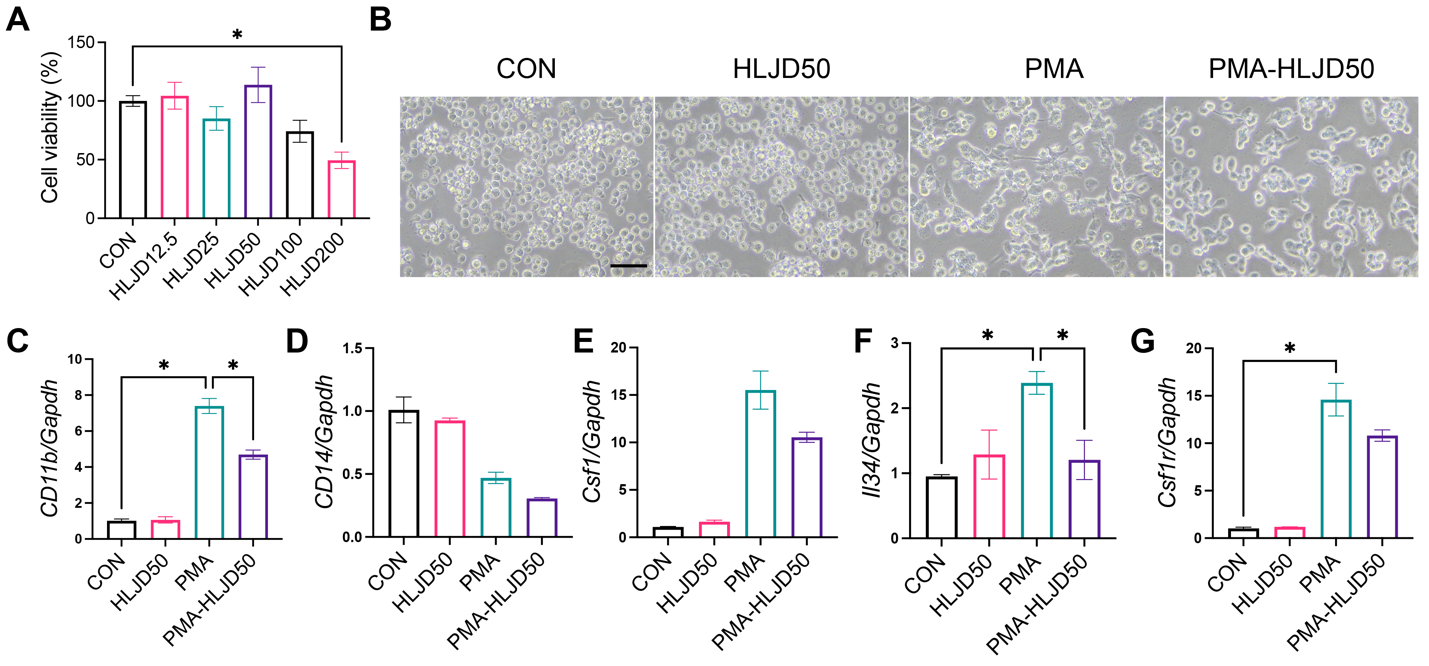


**Fig S1**. HLJD inhibited PMA-induced differentiation of THP-1 monocytes into macrophages. (**A**) Cell viability of THP-1 cells after exposure to different doses of HLJD (12.5/25/50/100/200 µg /ml) at 37 ̊C for 24 hours. (**B**) Representative images of THP-1 cells treated with 80 ng/ml of PMA with or without HLJD at the concentration of 50 µg /ml (Scale bar, 50 μm). (**C-G**) Relative mRNA expression of *CD11b*, *CD14*, *Csf1*, *Il34* and *Csf1r* normalized to Gapdh. At least three independent experiements were conducted. Data are shown as mean ± standard error of the mean. **P* < 0.05.


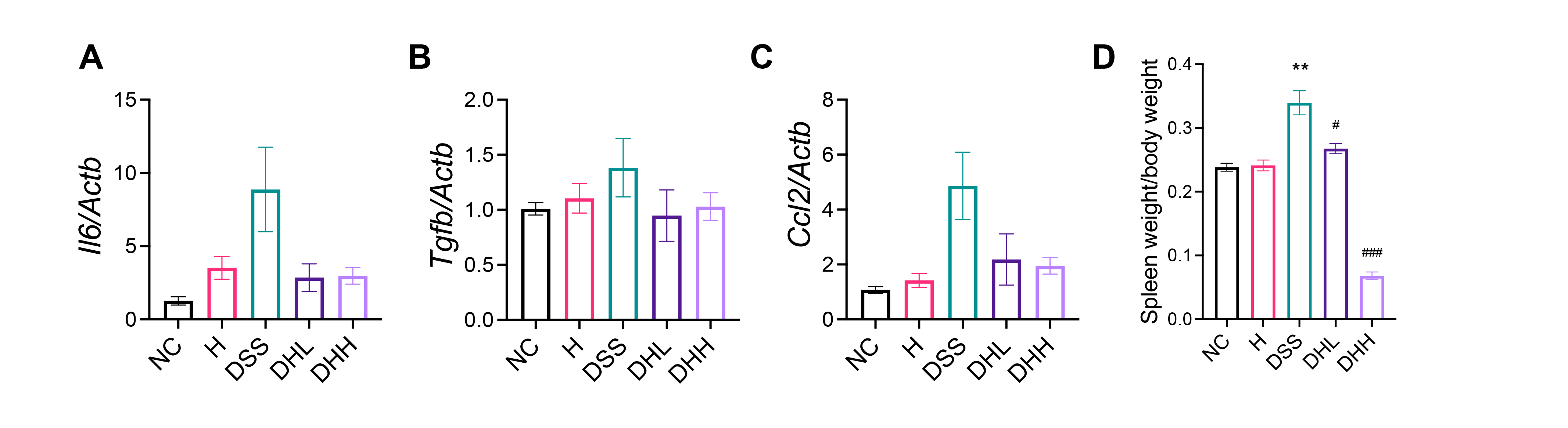


**Fig. S2** Spleen weight and relative mRNA expression of *Il6*, *Tgfb* and *Ccl2* normalized to *Actb*. (**A-C**) Relative mRNA expression of *Il6*, *Tgfb* and *Ccl2* normalized to *Actb* (n=6-9). (**D**) Spleen weight percentage (n=11). NC, normal control group; H, HLJD at the dose of 2 g/kg daily by oral gavage; DSS, mice given 1% DSS five days a week for 8 weeks; DHL and DHH, mice given 1% DSS were treated with HLJD at 2 g/kg and 4 g/kg daily by oral gavage, respectively. Data were expressed as mean ± SEM. ** *P* < 0.01 *vs.* NC; # *P* < 0.05, ### *P* < 0.001 *vs.* DSS.


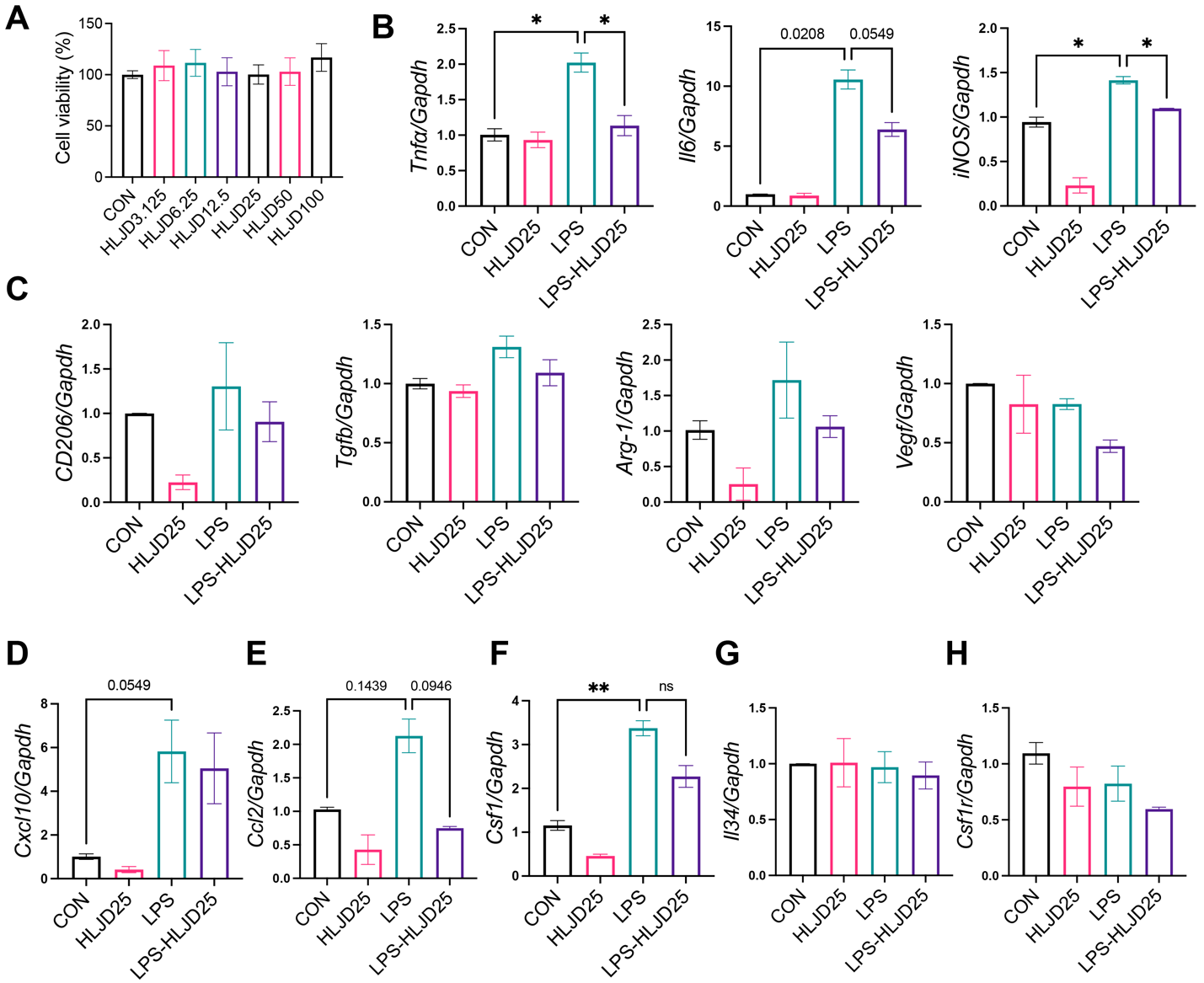


**Fig S3**. HLJD inhibited LPS-induced M1 phenotype in RAW264.7 macrophages. (**A**) Cell viability of RAW264.7 cells after exposure to different doses of HLJD (3.125/6.25/12.5/25/50/100/200 µg /ml) at 37 ̊C for 24 hours. (**B**) Relative mRNA expression of *Tnfa*, *Il6* and *iNOS* normalized to *Gapdh*. (**C**) Relative mRNA expression of *CD206*, *Tgfb*, *Arg-1*and *Vegf* normalized to *Gapdh*. (**D-H**) Relative mRNA expression of *Cxcl10*, *Ccl2*, *Csf1*, *Il34* and *Csf1r* normalized to *Gapdh*. At least three independent experiements were conducted. Data are shown as mean ± standard error of the mean. *p < 0.05, **p < 0.01.
